# Etiologic Framework for the Study of Neurodegenerative Disorders as Well as Vascular and Metabolic Comorbidities on the Grounds of Shared Epidemiologic and Biologic Features

**DOI:** 10.3389/fnagi.2016.00138

**Published:** 2016-06-13

**Authors:** Jesús de Pedro-Cuesta, Pablo Martínez-Martín, Alberto Rábano, María Ruiz-Tovar, Enrique Alcalde-Cabero, Miguel Calero

**Affiliations:** ^1^Department of Applied Epidemiology, National Center for Epidemiology, Carlos III Institute of HealthMadrid, Spain; ^2^Consortium for Biomedical Research in Neurodegenerative Diseases (CIBERNED), National Institute of Health Carlos IIIMadrid, Spain; ^3^Alzheimer Disease Research Unit, CIEN Foundation, Queen Sofia Foundation Alzheimer CenterMadrid, Spain; ^4^Chronic Disease Programme, Carlos III Institute of Health, MajadahondaMadrid, Spain

**Keywords:** epidemiological patterns, etiology of conformational protein deposits, templating underlying risk/progression, disease induction vs. transmission in amyloid, multidisciplinary research overlaps

## Abstract

**Background**: During the last two decades, protein aggregation at all organismal levels, from viruses to humans, has emerged from a neglected area of protein science to become a central issue in biology and biomedicine. This article constitutes a risk-based review aimed at supporting an etiologic scenario of selected, sporadic, protein-associated, i.e., conformational, neurodegenerative disorders (NDDs), and their vascular- and metabolic-associated ailments.

**Methods**: A rationale is adopted, to incorporate selected clinical data and results from animal-model research, complementing epidemiologic evidences reported in two prior articles.

**Findings**: Theory is formulated assuming an underlying conformational transmission mechanism, mediated either by horizontal transfer of mammalian genes coding for specific aggregation-prone proteins, or by xeno-templating between bacterial and host proteins. We build a few population-based and experimentally-testable hypotheses focusing on: (1) non-disposable surgical instruments for sporadic Creutzfeldt-Jakob disease (sCJD) and other rapid progressive neurodegenerative dementia (sRPNDd), multiple system atrophy (MSA), and motor neuron disease (MND); and (2) specific bacterial infections such as *B. pertussis* and *E. coli* for all forms, but particularly for late-life sporadic conformational, NDDs, type 2 diabetes mellitus (T2DM), and atherosclerosis where natural protein fibrils present in such organisms as a result of adaptation to the human host induce prion-like mechanisms.

**Conclusion**: Implications for cohort alignment and experimental animal research are discussed and research lines proposed.

## Introduction

During the last two decades, protein aggregation at all organismal levels, from viruses to humans, has emerged from a neglected area of protein science to become a central issue in biology and biomedicine (Villar-Piqué and Ventura, 2012) The expanding knowledge in the field has particularly benefited our understanding of neurodegenerative disorders (NDDs). At the present time, it is widely acknowledged that *templating* (the basic mechanism generating protein misfolding, aggregation, deposit, and cell-to-cell transfer of certain pathogenic proteins) would be able to explain many features of the subclinical course and clinical manifestations of NDDs (Warren et al., 2013). Transcellular disease propagation of the pathogenic protein along existing anatomic structures (neural networks) would also explain the progression from the varied clinical patterns of NDDs (denoted as phenotypic heterogeneity) to a phenotypically converging, similar status, where a wide loss of cognitive, motor and sensory functions occur (Warren et al., 2013).

In a prior article, we selected 11 neurodegenerative conditions defined by clinical, neuropathological and biochemical features expressing different sets of single or combined misfolded protein deposits, and described life-course-related epidemiologic features (age-specific incidences and disease risk factors or disease progression predictors), thereby allowing for a meaningful, framed and unifying view of each disorder and its post-mortem biochemical signature (de Pedro-Cuesta et al., 2015). At a second step, we used a modified definition of the driver concept proposed by Sutherland to identify risk factors of specific proteins affecting different NDDs (Sutherland et al., 2011) and, having in mind the common amyloid nature of the pathogenic proteins involved, we identified selected traits of the epidemiology of sporadic, protein-associated, i.e., conformational, NDDs (sCNDD) useful for interpreting epidemiologic findings and relationships of the entities listed in Table 1 (Gunnarsson et al., 1996; Marmorstein et al., 2002; Peng et al., 2005; Irwin et al., 2013; Prusiner et al., 2015; de Pedro-Cuesta et al., 2016). In this third article we face a cumulative etiologic approach taking into account recent experimental data on transmissibility of multiple system atrophy (MSA; Prusiner et al., 2015).

**Table 1:** **Modified from de Pedro-Cuesta et al. (2016) Main biochemical, epidemiologic and other features of sCNDDs and other sporadic human amyloid disorders involved in the proposed etiologic framework**.

Entity or neuropathologically related entities	Main protein deposit	Reported outbreaks		Annual incidence per million person-years or prevalence at death^†^		M/F ratio
				Sporadic	Genetic
Creutzfeldt-Jakob disease	APrP	vCJD	UK, Ireland, France, Spain	1	0.1	1.1/1
Amyotrophic lateral sclerosis (ALS) and	Ubiquitin, MAPT (tau),	ALS	Skaraborg county (Sweden;	10	1	1.5–2/1
frontotemporal dementia (FTD)	SOD, TDP-43/FUS		Gunnarsson et al., 1996).
			US human growth hormone treated cohort (Irwin et al., 2013)
Parkinson’s disease (PD),	α synuclein	–	–	100	10	1.5–2.5/1 for PD
Lewy body disease (LBD) and multiple system atrophy (MSA)
Alzheimer’s disease	β amyloid, Tau	–	–	1000	100	0.92/1
Late age-related macular degeneration (AMD)	EFEMP1 wild-type (Marmorstein et al., 2002)		–	1000	Unknown	0.95/1
T2 diabetes mellitus (T2DM)	Langerhans Islet peptide	–	–	–	–	–
Sporadic cerebral amyloid angiopathy	β amyloid wild-type	–	–	–	–	–
Senile systemic amyloidosis (SSA), heart failure and myocardial infarction, aortic aneurism, narrow spinal channel	Transthyretin wild-type	–	–	–	25% ≥85 years^†^	–
Medin arteriopathy (Peng et al., 2005)	Lactadherin	–	–	–	–	–

Transmission among individuals proven for CJD and PD, not for MSA*. *From PD patient to fetal grafted cells. Brain extracts of MSA cases transmitted α synuclein proteinopathy to TgM83^+/–^ mice (Prusiner et al., 2015). ^†^ For SSA.

Accordingly, the purpose of this work was: (a) to complement and anchor the interpretation of each *driver* with selected results of clinical or animal-model research; (b) to propose etiologic theory pointing to basic mechanisms and specific causal hypotheses testable by study designs, for epidemiologic or experimental research; and (c) in line with recent initiatives, to propose public health developments.

## Search Strategy and Selection Criteria

References for this review were identified by searches used in prior articles (de Pedro-Cuesta et al., 2015, 2016), by diverse searches of MEDLINE with limits from 1995 to September 2015, relevant for entities on study, and reference lists from relevant articles. Reports in MEDLINE in all languages, using each of the following diagnostic search terms combined with etiology, i.e., dementia, Creutzfeldt-Jakob syndrome, motor neurone disease (MND), amyotrophic lateral sclerosis (ALS), fronto-temporal dementia (FTD), MSA, Parkinson’s disease (PD), Lewy body disease (LBD), Alzheimer’s disease (AD), rapid progressive dementia, age-related macular degeneration (AMD) and Huntington’s disease (HD). There were no language restrictions. Driver specific reference lists were built. The final reference list consisted of a compendium of an approximately twice the size of full-text consultation work. As a literature review based proposal, the study does not require ethical assessment.

## Data Supporting Amyloid-Related Etiology According to Specific Drivers and Succinct Theory

### Driver 1. Low Age-at-Exposure Related Susceptibility to Environmental Exposure Effects

Driver 1 builds on the following specific epidemiologic observations: (a) age at first major whooping cough epidemic and PD incidence in Iceland; (b) bovine spongiform encephalopathy and variant Creutzfeldt-Jakob disease (vCJD); (c) age at first human growth hormone (hGH) treatment and accidentally transmitted CJD (atCJD) in the UK; and (d) risk of sporadic Creutzfeldt-Jakob disease (sCJD) from routine surgery followed by a ≥20-year latency period in Denmark and Sweden.

When fitted to a linear model, risk of PD increased with age at first whooping cough epidemic, a proxy of median age at BP infection (de Pedro-Cuesta et al., 1996). The susceptibility function for vCJD followed an inverted V shaped profile peaking at approximately 10 years. For sCJD, we observed that surgery at juvenile age (<30 years) yielded the highest risk (OR 12.80; 95% CI 2.56–64.0). Associations between developmental factors and risk of AD (Savica et al., 2013) and PD (Barlow et al., 2007) might support driver 1 validity for late-life sCNDD (for a review see de Pedro-Cuesta et al., 2016).

#### Complementary Findings

- Neuropathologically proven AD associated with atCJD has been reported in a young patient who received a dura mater graft (Preusser et al., 2006).
- A significantly high incidence of MND (based on three cases) has recently been described in a young cohort of hGH recipients in the USA (Irwin et al., 2013).
- Potential host-to-cell induction of α-synuclein degeneration from patients diagnosed with PD who received fetal/embryonic tissue grafts, and disease induction by seeding in the α-synuclein and tau mouse models, supported the inference of induction of late-life sCNDD in fetal cells (Luk et al., 2012b; Morales et al., 2012; Olanow and Brundin, 2013).
- Age-at-exposure related effect is a common characteristic of neurodegeneration induced by neurotropic agents (Fishman et al., 1985). In a 1-methyl-4-phenyl-1,2,3,6-tetrahydropyridine (MPTP) parkinsonism model, selective nigral neuronal toxicity in laboratory animals was highest for the older rats and macaques (Langston et al., 1987). This was attributed to age-related neuronal pigmented deposit (relevant for accumulation of metabolic residue and advanced glycation end-products), which increases with age.
- Intracerebral injection of brain extracts containing aggregated α-synuclein into young, α-synuclein-transgenic mice stimulates the formation of α-synuclein lesions in the host (Luk et al., 2012a; Mougenot et al., 2012).
- Experiments on different neurodegenerative disease models supporting prion-like mechanisms (Jucker and Walker, 2013): in seeding experiments with APP mice, the age of the host at the time of inoculation can be a strong determinant of the result (Jucker and Walker, 2013). A similar feature is displayed by the male-mouse castration model of PD, which is only efficient when castration is performed on 4- to 6-week-old mice (Khasnavis et al., 2013).
- MSA, an α-synucleinopathy, has recently been transmitted in cell and mouse models (Prusiner et al., 2015).

#### Theory

Early- and midlife proteinopathies and late-life sCNDD could be induced or modulated by early-age exposures, regardless of anatomic organs for contact and following a log-linear age-related function. Mimicking age-at-exposure related effects in animal models might be essential for supporting the biological plausibility of epidemiologic observations.

### Driver 2. Tridimensional Pattern: Direct Relationships between Age at Peak Incidence (or Age at Clinical Onset), Incidence Magnitude, and Course (Clinical Disease Duration)

The driver, described on the basis of sCJD, FTD, PD, LBD, AD, and AMD data suggest that:

(a) the duration of the subclinical and clinical courses are related;
(b) low-incidence sCNDD progress rapidly, and those with highest incidence progress slowly; and,
(c) agents (including modifiers) generating aggressive forms might be less frequent or require more susceptibility than those lying behind less severe forms which may display high attack rates, i.e., they will affect large proportions of the population.

#### Complementary Findings

**Figure 1:**
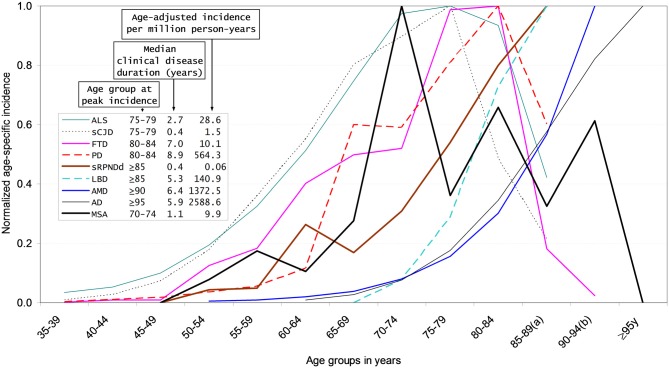
**Normalized age-specific incidence, incidence per million and survival for selected neurodegenerative disorders (NDDs).** Modified from de Pedro-Cuesta et al. (2015). Normalized age-specific incidence, age-adjusted incidence, and median clinical disease duration of different sporadic protein-associated neurodegenerative disorders (sCNDDs), obtained either from reported data [amyotrophic lateral sclerosis (ALS), personally modified by Fang F, sporadic Creutzfeldt-Jakob disease (sCJD)] or from registries [sporadic rapid progressive neurodegenerative dementia (sRPNDd) notified as suspected sCJD in Spain for 1995–2011, obtained from the Spanish CJD surveillance registry] and obtained from authors for multiple system atrophy (MSA). References for Figure 1 (Granieri et al., 1991; Gao et al., 1998; Baldereschi et al., 2000; Will et al., 2000; Benito-León et al., 2004; Pocchiari et al., 2004; de Lau et al., 2004; Fang et al., 2009; Chen and Lai, 2010; Steenland et al., 2010; Owen et al., 2012; Bjornsdottir et al., 2013). (a) 85–89 years is equivalent to 85 years and older for sCJD, ALS, Lewy body disease (LBD), Parkinson’s disease (PD), and sRPNDd; (b) 90–94 years is equivalent to 90 years and older for age-related macular degeneration (AMD) and frontotemporal dementia (FTD).

- Age at peak age-specific incidence appears to be related to protein propagation, as supported by observations of familial AD, where mean ages at onset are determined by the Ab40/42 ratio (Duering et al., 2005).
- Prion models suggests that a lower prion protein inoculum correlates with a longer incubation period (Gravenor et al., 2003). Slow subclinical and clinical progression might correspond to long incubation period from low inoculum exposures in experiments with animal models of prion diseases (Gravenor et al., 2003).
- Acute toxic effects followed by residual effects might better fit MPTP model patterns (Langston et al., 1987) than those suggesting a persistently active etiologic mechanism or multiple hits across the life course.
- After request to authors of recent surveys (Bjornsdottir et al., 2013; Caslake et al., 2014) and positive answer for Icelandic data, normalized reported age-specific incidence and median age-at-onset of MSA in Iceland where plotted at a reported figure for other CNDDs (de Pedro-Cuesta et al., 2015). The MSA incidence curve fitted curves and peak values intermediate between those for sCJD and PD (Figure 1; Granieri et al., 1991; Gao et al., 1998; Baldereschi et al., 2000; Will et al., 2000; Benito-León et al., 2004; de Lau et al., 2004; Pocchiari et al., 2004; Fang et al., 2009; Chen and Lai, 2010; Steenland et al., 2010; Owen et al., 2012; Bjornsdottir et al., 2013; de Pedro-Cuesta et al., 2015).

#### Theory

This driver might point to two distinct etiologic sCNDD forms differentiated by rapid progression, such as sCJD, ALS, and some sporadic rapidly-progressing neurodegenerative dementias (sRPNDds) with peak onset at age 70–75 years, and disease duration <3 years and late-life sCNDD with later age at onset and slower progression. When links with vascular lesions or vascular risk factors (VRF) are considered, sCJD, LBD, AD, and AMD would present and perhaps share vascular protein deposits: this would not, however, apply to FTD, PD and MSA, which are epidemiologically less closely related to VRF. Type 2 diabetes mellitus (T2DM) and vascular disease would be particularly seen in sCNDD (including sRPNDds) with incidence peaking after age 85 years. Prion strains and diverse entry routes would determine the large variation of age at onset in hGH-related atCJD, vCJD, and sCJD. High incidence of late-onset sCNDD might correspond to high attack rates from ubiquitous or widespread, tiny or low inoculum exposures and the opposite or a combination (low incidence, high inoculum exposures) for midlife-onset sCNDD, i.e., earlier-onset sCJD, ALS and earliest-onset sRPNDd may share prion-like entry or spread mechanisms.

### Driver 3. Shared, Age-at-Onset Related, Genetic Risk Factors

We postulate that driver 3 reflects the effect of different genes, which, either separately or by interaction, determine the excess risk of several sCNDD, such as AD, CJD, and LBD (Wilson et al., 1994; Zende et al., 2013). For instance, positive findings in sCJD and AD for *APOE*ε4, CALHM1 and BACE1 polymorphisms underscore the interplay between APP, Aβ oligomers, ApoE, PrP, and BACE1 in sCJD and AD, and suggest that aging may partly modulate disease pathologies through these key players (Calero et al., 2012a,b).

#### Complementary Findings

- It is accepted today that the animal models which best reproduce atherothrombotic disease and AD are those based on ApoE-knockout mice (Zaragoza et al., 2011).
- Diverse reviews on APOE gene effects show that the ApoE4 allele is linked to moderate excess risk for arterial hypertension and ischemic heart disease (Haan and Mayeda, 2010), intracerebral and lobar bleeding (Biffi et al., 2010), coronary heart disease, stroke, peripheral artery disease, and diabetes mellitus (Eichner et al., 2002).
- The common presence of APOE, BACE1 (and other genes, as suggested by total genome studies) linked to βA and other proteins calls for a role for these or other genes in a selected group of sCNDD, as well as in atherosclerosis.
- sCJD was initially misdiagnosed 4% of times as a vascular, non-autoimmune disease, including stroke (Paterson et al., 2012), perhaps indicating the presence of a vasculopathy in sCJD.

#### Theory

APOE4 interacts with etiologic environmental factors for several disorders, i.e., sporadic AD, LBD, AMD, vascular dementia, atherosclerosis, or other forms of vascular disease such as heart failure, myocardial infarction, senile systemic angiopathy, and aortic aneurism. We also hypothesize that shared susceptibility genes for selected sCNDD and atherosclerosis mediate the effects of single pluripotential exposures, i.e., due to the same inoculum, or generated by the same mechanism. High-risk birth cohorts might share excess risk for several conformational disorders.

### Driver 4. Personal Risk Factors

**Figure 2:**
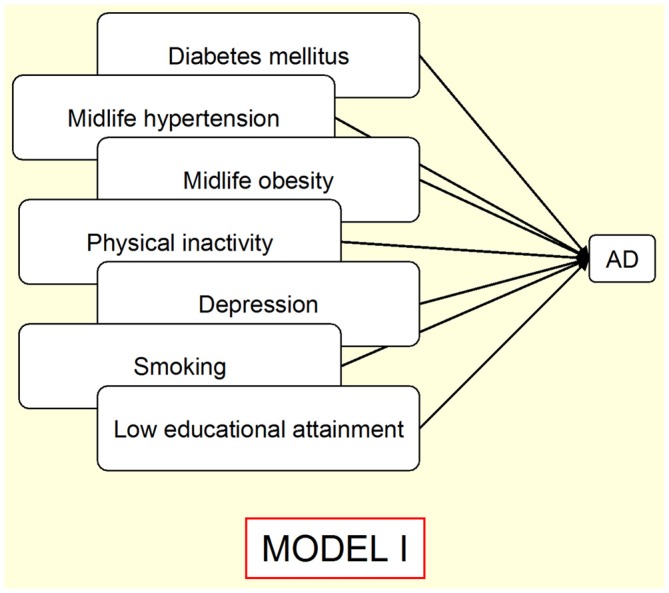
**Outline of a proposed Public Health Prevention Model I for a single neurodegenerative disorder (Norton et al., 2014), e.g., Alzheimer’s disease (AD).** Overlaps indicate risk-factor associations. Arrows indicate assumed direct causality.

This driver is related to a set of multiple variables, such as clinical signs and symptoms (diagnoses), behavioral patterns (health-related habits), and educational factors, which constitute the group of well-established associations with specific sCNDD. When discussing driver 1, reference was made to some personal factors potentially due to an age-at-exposure related effect, fitting under the umbrella of developmentally-related effects, i.e., education in AD, cranial perimeter in north-Korean women, or early symptoms, such as scoliosis or constipation in PD (Stern et al., 2012). This multifaceted and disperse panorama might be too wide for a satisfactory etiologically oriented approach to sCNDD. Insofar as the focus is AD or dementia, however, the driver notion constitutes the best structured etiologic and public health approach. A recent review addressing arterial hypertension, T2DM, hypercholesterolemia, physical activity, depression educational attainment, and smoking history (all personal factors) and risk of AD emphasized the non-independent nature of such factors (Norton et al., 2014). Correcting for dependence, the authors estimated that, assuming causality underlying the associations, the AD attributable proportion reduced by a control of 10% of risk factors per decade would be 8.3% worldwide in 2050 (Norton et al., 2014). When these risk factors were considered with regard to other sCNDD, the established association patterns were weaker. For instance, no excess risk for PD from T2DM was observed, when PD onset was controlled for Simon et al. (2007) and Palacios et al. (2011). In contrast, other studies reported modest increases (Schernhammer et al., 2011; Sun et al., 2012). We believe that the outline shown in Figure 2 (Norton et al., 2014) illustrates the state of art in public health prevention of sCNDD (entity-specific and mainly based on type-4 drivers). We propose that a complementary etiologic overview of sCNDD from personal factors should incorporate goals and interpretations from other drivers, and driver 1 in particular, for late-life sCNDD, as illustrated by a potential multiple outcome that combines (though not necessarily in the same individual) protein deposits generating sCNDD, T2DM as a result of Islet amyloid peptide (IAPP), and vascular wall lesions from amyloid deposits such as Aβ, lactadherin (Peng et al., 2005) and transthyretin (TTR; Coelho et al., 2013).

#### Complementary Findings

- There is increasing biologic evidence supporting the contention that fibrillar amyloid beta protein present in atheroma promotes atherogenesis (Medeiros et al., 2004).
- Bacterial vectors may transport inclusion bodies having amyloid-like properties able to seed their soluble counterparts and induce cytotoxicity in eukaryotic cells (for a review see Wang, 2009).
- A growing body of evidence suggests that platelet activation can mediate angiopathy in AD (Zhang et al., 2013). Medin-deposits, i.e., lactadherin, underlie age-associated arterial lesions (Peng et al., 2005).
- Colocalization studies of IAPP and Aβ in islet amyloid in type 2 diabetic patients, and Aβ deposits in brains of patients with AD show heterologous seeding between IAPP and Aβ, a phenomenon that may represent a molecular link between T2DM and AD (Oskarsson et al., 2015).

#### Theory

**Figure 3:**
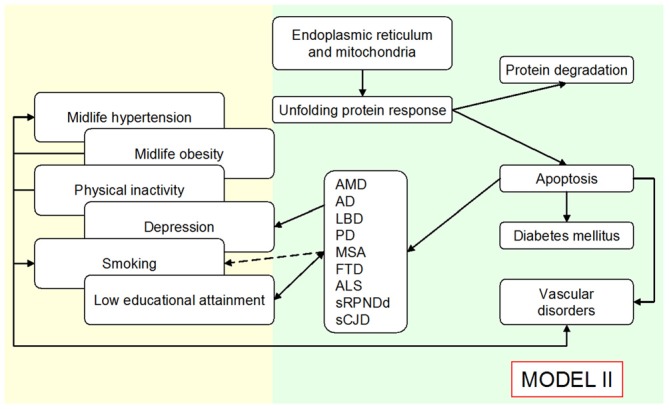
**Outline of an expanded Public Health Prevention Model I to Model II, assuming an age-at-onset related continuum for various late-age NDDs, and that the unfolded protein response explains—at least in part—the reported associations for diverse conformational neurodegenerative, vascular degenerative and metabolic disorders (type 2 diabetes mellitus, T2DM).** Modified from de Pedro-Cuesta et al. (2016).

The view based on personal factors and the amyloid perspective on sCNDD suggests that a considerable part of the associations with late-life sCNDD, atherosclerosis, and T2DM might conceal confounding or reverse association (early manifestations of subclinical disease). This view, if confirmed, might complement the rationale for prevention of sCNDD, T2DM, and part of atherosclerosis, where measures for control of shared etiologic mechanisms underlying sCNDD, T2DM, and various angiopathies are proposed. Driver 4 contribution to theory is summarized as a potential switch of the roles attributed to a direct relationship between risk factors and sCNDD in Figure 2, to those outlined in Figure 3. Most of these associations are in part interpreted as confounding or reverse causality, and direct causality is assigned to a higher level shared by defined amyloid disorders.

### Driver 5. Environmental Risk Factors

As a relevant part of our hypothesis we selected invasive medical procedures (mainly surgery) as a risk factor for sCJD and BP infection as a risk factor for PD.

#### Invasive Medical Procedures and Risk of Midlife sCNDD

The hypothesis of a surgical (and blood-transfusion-related) risk of sCJD is based on a few reports on CJD among patients exposed to neurosurgical instruments used on a patient with subclinical or clinically manifested CJD, and on observations from a register-based population case-control study on routine hospital surgery followed by a lag of over 20 years, with retina surgery being associated with sCJD, on the basis of three cases with a mean latency of 11 years (de Pedro-Cuesta et al., 2011). Aside from one Italian study (Puopolo et al., 2011), however, confounding by surgically-linked blood transfusion was not controlled for, and no positive association between neurosurgical procedures and CJD has been shown. Inconsistent findings for risk from blood transfusion have been reported.

#### Complementary Findings

- In 1994, infectivity from tissues such as liver and lung was shown in CJD mouse models (Brown et al., 1994). Subsequently, transmission of prion disease from tissue remnants adhered to surgical instruments was confirmed (Weissmann et al., 2002).
- Injection of mouse brain extract containing Aβ seeds (similar to those found in the AD brain) into the peritoneal cavity of mice genetically engineered to develop amyloid plaques (murine model of AD) accelerates Aβ deposition in the host brain (Eisele et al., 2010).
- βA deposition can be induced by injection of AD brain extracts into animals, which, without exposure to this material, would never otherwise develop these alterations (Morales et al., 2012).
- An independent line of evidence builds on seeding experiments from curli for *E. coli* and other organisms in Amyloidosis A (Lundmark et al., 2005).
- The long incubation suggested for sCJD from routine surgery undergone at high-susceptibility ages would suggest that such mechanisms may only be relevant for rapid-course sCNDD, so that the latency period for late-life NDDs might exceed the human life course.
- The increased resistance to protein degradation in amyloid may provide some consistency with prion theory.
- Recently some patients treated with cadaveric hGH who subsequently developed iatrogenic CJD also showed evidence of βA deposition in their pituitary glands (Jaunmuktane et al., 2015; Frontzek et al., 2016).
- Brain extracts of MSA cases, in general aged at onset ≤75 years, transmitted an α-synuclein proteinopathy to TgM83+/− mice being the prionic nature of some α-synuclein recognized (Prusiner et al., 2015).

#### Theory

On the basis of register-based epidemiologic studies on surgical risk of sCJD suggesting age-related susceptibility, the suggested associations of ALS and incidence peaking at a similar age, we propose that sCJD, ALS and some sRPNDds and MSA with onset below age 75–79 years are transmitted by routine surgery and transfusion of blood or blood derivatives at juvenile age.

#### Host-Adapted Human Pathogens. BP and Late-Life sCNDD

The association between age at first major whooping cough outbreak (MWCO) and PD constitutes an important observation in Iceland, reinforced by negative results for birth-cohort effects in continental populations. Considered as a quasi-experiment, it is consistent with the high prevalence and incidence of PD among the Faroe Islanders and Greenland Inuit (Wermuth et al., 2002, 2008). The excess risk of PD as a long-term biological effect of BP infection was attributed to Pertussis toxin (de Pedro-Cuesta et al., 1996). Since PD shares protein deposits (for a review see Sutherland et al., 2011 ) with MSA, LBD, and AD, BP infection in genetically susceptible young individuals might be proposed as an environmental driver for late-life sCNDD such as MSA, PD, LBD, AD, and AMD.

#### Complementary findings

- BP has historically been harbored by human populations and its dynamic still remains uncontrolled by vaccines. Despite the fact that undesired protein aggregation can have negative consequences for the cell, functional aggregation-prone regions and aggregates are essential for life. For example, in some enterobacteria, amyloid aggregates appear to mediate bacterial adhesion, biofilm development, and host invasion (de Groot et al., 2012). Human coexistence with commensal or pathogenic bacteria such as BP might have led to the interaction of bacterial aggregated proteins with aggregation-prone sequences from the host, resulting in xeno-templating.
- A mutation in the PS2 gene (M239V) neutralizes the toxicity mediated by the Pertussis toxin, suggesting that this toxin shares pathological mechanisms with some AD-mutant variants (Abe et al., 2004).
- Biological agents acting in the distant past may have changed as a result of human interventions, such as vaccination (Cummings et al., 2004).
- Horizontal gene transfer research on the Tohama I strain of BP dating back to 1950 may provide source information for understanding the pathobiology of BP and the potential effect in Japanese cohorts currently aged 70 years (Preston, 2005), particularly if curli can be identified in such strain.
- The rationale suggested for *E. coli* (see “Driver 7. Invariant ratio of sCNDD Incidence/Genetic CNDD Incidence, Across Entities” Section) would also be valid for BP. Seeding of naturally occurring protein fibrils from BP, as experimentally observed with curli from *E. coli* (Lundmark et al., 2005), might be hypothesized as biological pluripotential mechanisms underlying BP infection as a causal factor of PD, AD, and diverse late-life sCNDD.
- Mechanisms for adaptation to humans seem to have occurred in the BP precursor, *B. pseudopertussis*, at an earlier time, taking place for BP by gene loss (Diavatopoulos et al., 2005). *B. pertussis* infection damages nasal ciliated epithelium, a structure which transfers uranium to brain along olfactory nerve bundles (Wilson et al., 1991; Ibáñez et al., 2014).
- The clear, saw-teeth, birth-cohort pattern seen for PD incidence and attributed to the periodic pattern of BP epidemics in Iceland suggests that a similar effect of agents, other than airborne BP epidemic agents, such as influenza or measles, is unlikely. However, a supplementary action through the gastrointestinal tract is not ruled out. The shared early loss of olfactory function and lesions described in sCJD, FTD, PD, and AD might point to a potential effect of BP infection on the clinical profile of sCNDD (Magerova et al., 2014).
- In addition to airborne agents, synthetic amyloid-like biomaterials and oral agents such as those described for vCJD or food have been proposed, given the high content in protein fibrils (Westermark and Westermark, 2010).
- In accordance with the induction of AApoAII amyloidosis by various amyloid fibrils, though best by mouse AApoAII(C) amyloid (Fu et al., 2004), the expected key active element mediated by the human microbiome might be a natural fibril genetically related to human pathology.

#### Theory

Human-adapted agents integrating human genome such as BP able to generate natural protein fibrils might generate different late-life sCNDD by pluripotential mechanisms in accordance with the individual susceptibility. BP may act through nasal and gastrointestinal epithelia. Systemic action across the endoplasmic reticulum (ER) and vascular endothelium might be plausible.

### Driver 6. Endoplasmic Reticulum Stressors or General Drivers

Protein folding is a basic mechanism regulating cell function and survival (Bernales et al., 2012). Variables related to the unfolding protein response (UPR) in ER and mitochondria, acting either as stressors or as a result of altered pathways might be epidemiologically captured, reflecting some of the most characteristic drivers of the expanded NDD paradigm. They may affect sCNDD, T2DM, and atherosclerosis potentially related with senile systemic angiopathy and cerebral amyloid-β (Aβ) angiopathy (CAA; linked to myocardial infarction and dementia or AD, respectively), with different underlying amyloid deposits, i.e., wild-type TTR, lactadherin, and beta amyloid. In accordance with the original general proposal, such drivers may affect membrane or secretory tissues such as those resulting in estrogen and testosterone deficits, and midlife VRF such as metabolic syndrome and T2DM. Relationships may be complex if some of the ER stress effects or causes constitute T2DM or sCNDD comorbidity, i.e., obesity.

#### Complementary Findings

- The role of endocrine factors in sCNDDs risk is supported by clinical and animal research. Since premenopausal oophorectomy is an iatrogenic factor, at least some associations of estrogen deficit may be considered to act outside the most susceptible lifetime points (driver 1), such as juvenile age.
- In addition to the estrogen deficit in dementia, AD pathology, T2DM, and atherosclerosis discussed elsewhere (de Pedro-Cuesta et al., 2016), an impact on synuclein-related sCNDD may be suggested, since some animal studies implicate a role for estrogen in protecting the nigrostriatal dopaminergic functions (Becker, 1990; Morissette and Di Paolo, 1993).
- However, estrogen therapy with esterified estrogen use in combination with progestin was recently linked to a 6-fold excess risk of PD (Lundin et al., 2014). While castration, conducted no later than at 4–5 weeks, makes the male-mouse model support the etiologic role of testosterone deficit in PD (Khasnavis et al., 2013), it may also convey insights into the driver-1 effect better than does andropause mimicry.
- The fact that some endocrine deficits, i.e., testosterone, are linked to risk of other endocrine disorders raises questions about a shared, unidentified etiologic cause affecting secretory systems.
- Complex links are suggested for T2DM: T2DM could be also taken as a general driver, since it is associated with risk of both FTD (Golimstok et al., 2014) and AD (Jayaraman and Pike, 2014), and at the same time constitutes an outcome of testosterone depletion (Kapoor et al., 2006; Lage et al., 2007).
- The relationship of ER stress and vascular wall pathology may be persistent, since increased ER stress was identified in atherosclerotic plaques linked to coronary syndrome (Myoishi et al., 2007).
- The UPR in ER may be affected by environmental insults and genetic modifiers which hamper biosynthesis of steroids, cholesterol and many lipids (Kaufman, 2002).
- We believe that the amyloid perspective may serve as a reference for etiologic thinking, where shared determinants of early amyloid deposits in myocardial or cerebral vessels, i.e., the tau/AD pathology (Chui et al., 2012) are plausible. Chronic endocrine disorders such as estrogen or testosterone deficits might not yet have been identified as conformational disorders.

#### Theory

Figure 3 depicts a hypothetical etiologic scenario modified from Figure 2, where different amyloid deposits shared by sCNDD, vascular disorders and a number of endocrine entities or functions, determine late-life human pathology as a result of ER stressors or poor UPR. As a complement, some endocrine factors of a different origin (i.e., oophorectomy or specific estrogen therapies) might also constitute risk factors for conformational disorders.

### Driver 7. Invariant Ratio of sCNDD Incidence/Genetic CNDD Incidence, Across Entities

This driver applies basically to genetic CNDDs determined by point mutations. The ratio, best known when defined by a proxy value, namely, the proportion of incident disease corresponding to familial forms, tends to increase due to the progressive improvement in the identification of such mutations, and may reach 25% in selected sCNDD. This feature, emphasized by Soto and Estrada (2008), has long been recognized, despite the fact that this proportion is higher in a few populations (Israeli Jews, or populations with genetic clusters, e.g., Slovenia for CJD). Similarly low proportions have been described for genetic vs. sporadic forms of T2DM. The proportion is less clearly perceived for AMD where mutated or wild-type EFEMP1 has been described, and atherosclerosis forms, such as SSA, heart failure and aortic aneurysm (Westermark and Westermark, 2010), where wild-type TTR deposits have been described but an incidence or case series reference for the sporadic form is lacking.

#### Complementary Findings

- The ecologic relationship between sporadic genetic forms may suggest a causal link between genetic CNDDs resulting from point mutations and the corresponding sCNDD forms. Since the predominant protein signature of a specific sCNDD frequently corresponds to that of its genetic CNDD form, one might speculate that, ontogenically speaking, sCNDD forms might be considered secondary to genetic ones in terms of numbers and biochemical similarity (Parcerisas et al., 2014).
- In a prior article (de Pedro-Cuesta et al., 2016), we proposed that the most relevant field of knowledge for driver 7 and assessment of the role of vectors in sCNDD might be gene transfer from human to bacterial human pathogens. A first key issue would be the potential of human sequences to make the hosted agent generate misfolded proteins, resulting in infective natural fibrils with a biochemical signature determined by the transferred genetic material. The hypothesis would require an impact on sporadic atherosclerosis of mutations other than those seen in the TTR gene and Notch 1 present in Cerebral Autosomal-Dominant Arteriopathy with Subcortical Infarcts and Leukoencephalopathy (CADASIL), but the high nonspecific potential of natural and synthetic protein fibrils to enhance the amyloid response during inflammation might explain cross-effects (Fu et al., 2004).
- In this essay, we provide an example of such a potential process which might have been identified, namely, that of *E. coli*. It has been estimated that more than 17% of the genetic material of *E. coli* (an enterobacterium hosted by humans; Lawrence and Ochman, 1997), whose curli, natural protein fibrils, have been used in animal models of A-amyloidosis by natural fibril-seeding, is the result of horizontally transferred, protein-coding DNA over several million years (Lawrence and Ochman, 1997; Lundmark et al., 2005).
- Since inflammation is required for *templating*, the role of *E. coli* might be mediated by gastrointestinal infections at a young age. In 2003, Braak and associates proposed that transneuronal transmission of pathologically altered α-synuclein from the enteric nervous system to the brainstem might occur in PD (Braak et al., 2003). Various heterogeneous, natural and synthetic amyloid fibrils induced AApoAII amyloidosis (Fu et al., 2004). This process could be compatible with a proportion of cases being determined by other mechanisms or entry sites such as the oral cavity (Olsen and Singhrao, 2015). In contrast, SB Prusiner suggests that the late onset of heritable NDDs, like their sporadic counterparts, may reflect a stochastic nature of prion formation (Prusiner, 2013).
- Based on epidemiologic and laboratory research, it has been proposed that several infectious agents are linked to different NDDs: *Nocardia sp*. and BP to parkinsonism (Kohbata and Beaman, 1991; de Pedro-Cuesta et al., 1996), and recent adeno-associated viral vectors provide excellent α-synuclein models of PD (Lindgren et al., 2012). Similarly, Herpes simplex virus type 1 (HSV-1), in conjunction with apolipoprotein E4 (ApoE4) from a murine acute infection model, has recently been proposed as a risk factor for AD (Burgos et al., 2006; Harris and Harris, 2015). A similar relationship has been suggested for fungal infection and AD or ALS (Alonso et al., 2015a,b; Pisa et al., 2015a,b). The field of potential vectors, if restricted to well-adapted human pathogens, remains yet undefined but many are already sequenced.

#### Theory

Curli from some human-adapted pathogens correspond to multiple potentially infective natural misfolded protein fibrils resulting from horizontal gene transfer (HGT) from agents well adapted to the human host, acting at young ages as pluripotential agents facilitating invasion, particularly during inflammatory conditions (infections). These mechanisms may be shared by some angiopathies and T2DM exhibiting a similar driver, as well as by multiple similar pathogenic processes among humans. An essential condition for bacterial agents being candidates for HGT is that they themselves or their close ancestors must have been well adapted to human hosts by integrating pathogenic DNA from different niches (gingival, nasal, respiratory mucosae or gut epithelium, and other). In essence, infection by *E. coli* or coexistent with *E. coli* as a part of the intestinal microbiome, and other well adapted human hosts carrying pathogenic human genetic material and natural protein fibrils might induce diverse sCNDD, T2DM, and atherosclerosis, following an age-at-infection pattern.

## Main Features of a Causal Hypothesis for Scndd, Atherosclerosis, and T2DM

We propose that sCNDD, T2DM, a relevant proportion of atherosclerosis, as well as other late-life endocrine disorders resulting in andropause and estrogen deficit, constitute organ-limited amyloid disorders, where conformational mimicry leads to misfolding of a limited number of proteins such as PrP, Langerhans Islet peptide, amyloid beta (Aβ), tau, α-synuclein, SOD1, FTP-43, medin, TTR, and wild-type EFMP1 (Marmorstein et al., 2002).

Initial biological mechanisms are rooted in human genomics. Eukaryotic DNA fragments implicated in genetic forms of late-life NDDs [mainly AP and PD] incorporated in the genome of potential commensal, symbiotic, and pathogenic microorganisms present in the human reservoir, i.e., *E. coli* and BP, constitute sequences resulting in pluripotential protein fibrils that are infective via the respiratory and gastrointestinal tract or by B lymphocyte presentation. Some amyloid proteins such as APrP may additionally act parenterally by contact with human misfolded protein fibrils, generating rapid course sCNDD, such as sCJD, ALS, and some sRPNDds. Disease progression and spread through the central nervous system would follow reported anatomic patterns. Early vascular-wall lesions determine the first steps in atherosclerotic plaque formation. Small-vessel and neuronal lesions frequently coexist. The same pathogenic mechanisms may underlie some endocrine disorders. Multiple degenerative disorders may share conformational pathophysiologic mechanisms linked to different, as yet unknown, UPR. Latency intervals might frequently encompass decades.

**Figure 4:**
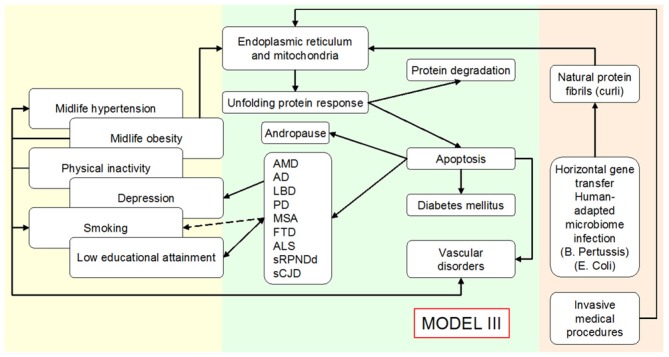
**Outline of an expanded Public Health Prevention Model II to Model III for various conformational neurodegenerative, vascular degenerative disorders and metabolic disorders (type 2 diabetes mellitus), assuming unfolded protein response acts as a consequence of at least two environmental causal mechanisms/agents, namely, infection/inflammation by human-adapted microbiome (examples for *Bordetella pertussis (BP)* and *Escherichia coli*) and invasive medical procedures**.

sCNDD epidemiologic patterns point to a high susceptibility in late-infancy and at juvenile ages. Risk and disease spread can be determined by the same factors, which constitute risk factors or biomarkers of disease progression, or both. ER stressors and UPR-related pathology, particularly late in life, may display complex relationships, i.e., as seen for VRF, late-life sCNDD, atherosclerosis, and T2DM. Incorporation of drivers 5,6, and 7 in the model seen in Figure 3, would result in a comprehensive etiologic model presented in Figure 4.

## Implications for Research

Components of the model seen in Figure 4 would have implications for cohort alignment and experimental animal research, as well as a for specific research outline proposals aimed to test fragments of the abovementioned hypothesis.

### Cohort Alignment

The present view of sCNDD reinforces the notion of a set of neurodegenerative processes in which continuity as a form of overlap, and competition—earlier and more lethal NDDs, T2DM or vascular disease removing persons at high risk of other less lethal NDDs—act on the age axis. Equivalent, observational, proof-of-principle studies would correspond to investigations aimed at confirming that NDDs which are clinically and epidemiologically close by median age at onset, disease duration, age-specific normalized incidence profile and genetic type of change, share environmental risk factors. The study of exposures in the first two decades of life would require approaches separated from that of the same exposures at later ages, with latency analyses being needed in both instances. Simultaneous approaches including two or more NDDs may be advantageous. Identification of selected populations by criteria targeted at optimizing ascertainment of both exposure and outcome, plus identification of randomly selected population controls are paramount.

### Experimental Animal Research

Implications of the driver notion from sCNDD epidemiology in experimental research appear to be less direct than in cohort alignment, requiring translation to biological inference. Different animal models have been developed to address various sCNDD, cardiovascular disease complications, and T2DM. Models in mice targeting AD, tauopathies, and ALS are mainly based on transgenic methodologies, with those for PD and synucleinopathies being both toxic and transgenic. Animal models may exhibit a number of important limitations, mimicking phenotypic variants of AD and other sCNDD^1^. The validity of human drivers, i.e., age-at-exposure related susceptibility, may be tested in toxic animal models of NDD.

In general, little attention has been paid to disease-non-specific animal models based on seeding of naturally produced amyloid fibrils, such as those generated by Sup35 from *Saccharomyces cerevisiae*, and curli from *E. coli* which exert amyloid-accelerating influences in experimental murine amyloidosis (Lundmark et al., 2005). In contrast, the validity of traditional approaches is extensively considered in disease-oriented media. A human driver-1-related, ApoE −/− mouse model, is that which best fits the study of arteriosclerosis and diabetes-accelerated arteriosclerosis (Zaragoza et al., 2011). A similar feature displays the male-mouse castration model of PD (Khasnavis et al., 2013). It would appear that the most valid NDD models are the genetically-modified, cell and animal models of AD, PD, FTD, and ALS, all of which are based on the mutations described in the familial NDD forms^2^. A research goal in the experimental model context might be the identification of key elements underlying an environmentally modified, potential effect of human genes, similar to that shown by laboratory experiments on NDDs, T2DM and atherosclerosis with traditional models.

Specific attempts to combine population and clinical cohorts/registries able to trace human events determining current disease might be a priority, particularly if: (a) such events can be identified by drivers; and (b) agents, similar to natural fibrils used in seeding models reproducing amyloid disease including NDDs, can be identified from historical sources, banks or libraries.

## Notions for Research Framework Development

### Epidemiology

Traditionally, neuroepidemiologists have been used to working with surveys or cohorts geographically settled close to their residence. As a novelty, assessment and alignment of cohorts for specific research purposes might be done for the first time with a high number of population or clinical cohorts gathered and offered as a potentially shared research resource^3^. On the basis of best clinical and epidemiologic practice, research into this avenue mighty advantageously address the etiology of and risk factors for sporadic forms of CJD, sRPNDd, MND, MSA, LBD, FTD, PD, AD, and AMD. This would require work on populations exposed to biological environments in specific conditions and with exposures measured under extremely favorable conditions for epidemiologic comparisons pertaining to registration and the time lag after exposure. Only a few populations in the world would allow for such field studies. To avoid misclassification of outcomes (subclinically affected controls), studies on the very old should be extremely cautious.

Possible factors to be weighted when considering favorable research environments are:

- Populations with registered data on both sRPNDd and sCJD tend to be large countries where CJD surveillance has been conducted for decades, and NDD- as well as CJD-free clinical controls have been registered.
- Medical procedures can be advantageously studied in countries with national health services that provide health care for the majority of their citizens.
- Controls randomly selected from annual population registries are paramount for studies on risk from medical procedures.
- Historically, populations exposed to agents producing point-source epidemics which are considerably separated in time and act on the young population, have been those that are geographically isolated with ancient native populations <200,000. A minimum size or pooled study would be required for substantial incidence counts of late-life NDDs. Study populations of this nature are to be found in Iceland, New Caledonia, San Miguel in the Azores (Aleixo-Dias et al., 1993), and a few other islands.
- In order to reveal the causal nature of specific associations, it might be advantageous to study the synergistic effects observed between environmental exposures and selected recognized genetic risk factors for different entities.

### Experimental Work

Research of this nature might encompass:

- Identification of potential agents (well adapted, small genome) with biological samples in potential vector libraries worldwide, in cases where such agents are implicated in NDD incident in high-risk cohorts;
- *In silico* identification of human genome fragments in vectors exhibiting curly or natural fibrils potentially involved in NDD induction, as seen from analysis of specific cohorts;
- Protein aggregate characterization in biological samples (bacteria, etc.), work with biofilms; and,
- development of animal models of defined or undefined amyloid disorders, based on the seeding of natural fibrils potentially involved in NDD induction.

## Author Contributions

JdP-C conceived and coordinated the study, analyzed literature, wrote first manuscript version. PM-M analyzed clinical literature and animal models reports, and contributed with comments. AR contributed with neuropathology literature and comments. MR-T revised aspects on prion disorders and provided comments. EA-C updated statistical calculations on incidences, and designed graphs. MC analyzed biochemical, genetic and molecular literature; revised in detail the manuscript and added comments. All authors read and approved the final manuscript version.

## Funding

Funding was received from the Consortium for Biomedical Research in Neurodegenerative Diseases (Centro de Investigación Biomédica en Red sobre Enfermedades Neurodegenerativas/CIBERNED) as part of the 2014–2015 annual budget of the CIBERNED 509-group, and from the Carlos III Institute of Health (PI12/00045). Mr. Alcalde received support from the ISCIII Field Epidemiology Program during 2014 and 2015. The study was partially supported by a grant from the EU Joint Program—Neurodegenerative Disease Research (JPND—DEMTEST (Spanish Health Research Fund, FIS PI11/03021)). The funders had no role in study design, data collection and analysis, decision to publish, or preparation of the manuscript. No author other than EAC received specific funding for this work.

## Conflict of Interest Statement

The authors declare that the research was conducted in the absence of any commercial or financial relationships that could be construed as a potential conflict of interest.

## Footnotes

^1^http://www.neurodegenerationresearch.eu/initiatives/jpnd-alignment-actions/animal-and-cell-models.

^2^http://www.neurodegenerationresearch.eu/initiatives/jpnd-alignment-actions/animal-and-cell-models.

^3^http://www.neurodegenerationresearch.eu/initiatives/jpnd-alignment-actions/longitudinal-cohorts/.
